# Effect of Laser Shock Peening Times on Low-Cycle Fatigue Properties and Fracture Mechanism of Additive TA15 Titanium Alloy

**DOI:** 10.3390/ma18245670

**Published:** 2025-12-17

**Authors:** Xu Pei, Sailan Wang, Zhaomei Xu, Zhouzhi Gu, Yuchun Peng, Pengfei Li

**Affiliations:** 1 Faculty of Mechanical and Material Engineering, Huaiyin Institute of Technology, Huai’an 223003, China; 2 School of Mechanical Engineering, Jiangsu University, Zhenjiang 212013, China; 3 Department of Smart Agriculture and Engineering, Wenzhou Vocational College of Science and Technology, Wenzhou 325006, China; pengyuchun@wzvcst.edu.cn

**Keywords:** laser shock peening, additive manufacturing, titanium alloy, fatigue life, fracture mechanism

## Abstract

This study investigates the effects of multiple laser shock peening (LSP) treatments on the low-cycle fatigue performance and fracture mechanisms of laser-melted, additive-manufactured Ti-6.5Al-1Mo-1V-2Zr (TA15) titanium alloy. The primary objective is to systematically evaluate how different LSP impact numbers (0, 1, and 2 impacts) enhance fatigue life and alter fracture behavior. Low-cycle fatigue life was determined via tensile-compression fatigue testing. Microfracture morphology was examined using scanning electron microscopy (SEM), surface residual stresses were measured by X-ray diffraction (XRD), and microhardness tests were conducted concurrently. Results indicate that LSP significantly enhances fatigue life: fatigue life increased by 2.34 times and 2.56 times after one and two LSP impacts, respectively, compared to the untreated state. As impact cycles increased, the microhardness of the material surface rose by 8.51% and 14.53%, respectively, with residual compressive stresses reaching −145 MPa and −183 MPa. Concurrently, LSP-2 treatment formed a refined microstructure featuring coexisting lamellar α and acicular martensite in the surface layer. This strengthening effect is attributed to LSP-induced surface residual compressive stress, grain refinement, and the resulting migration of fatigue crack initiation from the surface to subsurface regions. These findings provide critical insights for optimizing fatigue-resistant designs of additively manufactured titanium alloy components.

## 1. Introduction

Ti-6.5Al-1Mo-1V-2Zr (TA15), as a typical near-alpha high-temperature titanium alloy, exhibits outstanding high specific strength [1], excellent thermal stability [2], and superior corrosion resistance [3]. These advantages have led to its widespread application in aerospace critical load-bearing structural components [4], such as airframe frames and aircraft engine compressor blades [5]. In recent years, as aircraft structural design continues to evolve toward lightweighting, integration, and functional consolidation [6], component geometric complexity has increased while in-service performance requirements have become increasingly stringent. This presents greater challenges to the reliability and service life of TA15 titanium alloy under long-term fatigue loading.

However, traditional forged TA15 components face problems such as large machining allowance, low material utilization, and difficulty in manufacturing complex structures [7]. Additive manufacturing technologies such as laser melting deposition (LMD) provide unprecedented freedom for manufacturing TA15 components with complex geometries [8], and are expected to achieve efficient, lightweight, and integrated forming of components [9,10]. Although additive manufacturing technology has significant advantages, its inherent rapid melting and solidification process can easily introduce defects such as pores and unfused parts into the components, and form coarse columnar crystal structures [11]. These defects and unfavorable structures often act as stress concentration points, significantly reducing the fatigue performance of the material [12,13], especially fatigue life, which is extremely sensitive to surface stress [14,15], which seriously restricts the application of additive manufacturing TA15 components under fatigue critical conditions. For example, the fatigue performance of Ti-6Al-4V samples produced by P. Edwards [16] and others using selective laser melting additive manufacturing technology is significantly lower than that of forged materials, which is due to the influence of multiple factors such as porosity, surface finish and residual stress; Tang et al. [17] found that pore defects are the primary cause of fatigue failure in directional energy deposition (DED)-processed Ti-6Al-4V. Fatigue crack initiation can be categorized into two main types: internal pores and subsurface pores. Fine grain areas (FGAs) are commonly observed around internal pores, whereas FGAs are absent around subsurface pores.

In order to improve the fatigue performance of metal materials, surface strengthening technology has been proven to be an effective way [18]. Among them, laser shock peening, as an efficient surface modification technology, introduces deep residual compressive stress and refined microstructure into the surface layer of the material through the plasma shock wave induced by high-power density laser [19,20], so as to effectively inhibit the initiation and propagation of fatigue cracks [21,22]. For example, Wu [23] et al. used ultrasonic shot peening (USP) to strengthen the TA15 titanium alloy prepared by LMD, and found that the fatigue behavior of the material increased by 200% and 43%, respectively, at the stress levels of 720 MPa and 760 MPa; Kumar [24] et al. used USP to perform surface nano-meterization treatment on Ti-6Al-4V alloy, and found that surface strengthening improved the yield strength and tensile strength of the material, but the ductility was slightly reduced; Tang et al. [25] employed the LSP system to systematically investigate the influence of incidence angle on the surface integrity and wear resistance of hole structures. They found that oblique laser shock treatment enhances surface properties at the cost of reduced affected layer thickness. The optimal incidence angle was determined to be 30°, yielding peak surface microhardness and residual compressive stress values of 633.5 HV1 and 517.4 MPa, respectively, with the friction coefficient reaching a minimum of 0.542.

Existing research has confirmed the beneficial effects of laser shock peening (LSP) in conventional titanium alloy manufacturing [26,27,28]. For instance, Fang [29] et al. investigated the improvement of microstructure and properties in Al-Mg alloys through LSP, finding that dual-pulse specimens with 4J energy exhibited reduced porosity to 0.01% and increased yield strength by 89% (257 MPa). Nemanja Kljestan et al. [30] observed that LSP surface integrity enhances the high-cycle fatigue strength of laser powder bed fusion-processed low-alloy ultra-high-strength steel, with beneficial compressive residual stress fields forming near the strengthened surface. S. Pradeep Kumar et al. [31] tested the high-temperature wear resistance of LSP-hardened stainless steel manufactured via selective laser melting (SLM), finding that LSP conditions consistently exhibited the lowest wear with a 17% reduction in wear rate, demonstrating LSP’s effectiveness in enhancing the high-temperature tribological properties of SLM 17-4 PH stainless steel; Qu et al. [32] subjected DH36 welded joints to LSP. Results showed that 7 J of energy treatment increased microhardness in the weld zone from 195 HV to 231 HV. Residual tensile stress on the sample surface was neutralized by LSP and replaced by residual compressive stress. The high-cycle fatigue limit of the samples improved significantly, with an increase of 14.34%.

However, existing research has primarily focused on the LSP strengthening effects of conventionally forged or cast titanium alloys, or has addressed LSP treatment of additive-manufactured titanium alloys using single process parameters. However, systematic investigations into the influence of LSP treatment cycles on the fatigue properties of additively manufactured TA15 titanium alloy remain insufficient, particularly lacking a comprehensive mechanism explanation integrating multidimensional information such as fatigue life, fracture morphology, microstructural evolution, and surface residual stress and hardness distribution. Furthermore, the evolution patterns of fatigue crack initiation and propagation under different LSP cycles, along with their correlation mechanisms with surface microstructural gradients and residual stress field distributions, remain unclear.

To this end, this paper systematically investigates the effects of different LSP cycles (0, 1, and 2 cycles) on the rotational bending fatigue life and fracture mechanism of laser-melted deposited TA15 titanium alloy. The objectives of this study are: to quantitatively evaluate the fatigue life enhancement effect of LSP cycles on additively manufactured TA15; to reveal the mechanism by which LSP alters fatigue crack initiation locations and propagation behavior through macro- and micro-fracture analysis; and to elucidate the structure-property relationship between LSP-induced surface microstructural evolution (e.g., grain refinement) and fatigue performance, combined with lateral microstructural observations. The findings will provide crucial theoretical foundations and experimental data for fatigue-resistant design and surface integrity control of additive-manufactured TA15 titanium alloy components.

## 2. Materials and Methods

### 2.1. Materials and Preparation Processes

**Figure 1:**
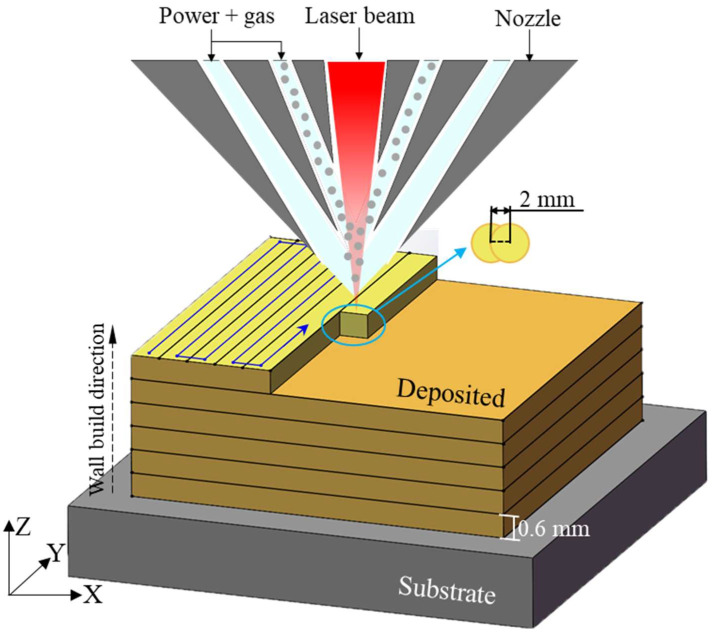
Schematic diagram of the sample prepared by the LMD process and the laser trajectory diagram.

Figure 1 illustrates the schematic diagram of the sample prepared using the LMD process. The LMD system comprises a control system, a fiber laser, and an inert gas. The TA15 sample was deposited layer by layer in an argon atmosphere with an oxygen content below 100 ppm. This study employed a YLS-2000 fiber laser (China, Nanjing) with a focused spot diameter of 4 mm to additively manufacture rectangular corrugated tube structures in an argon atmosphere. Laser power was set at 2400 W, scanning speed at 10 mm/s, and layer height at 0.6 mm per deposition layer. Key manufacturing parameters are listed in Table 1. The substrate material was a 60 mm thick forged TA15 titanium alloy with spherical powder particles ranging from 53 to 150 μm in diameter. Its chemical composition is detailed in Table 2. This alloy exhibits high strength, toughness, and high-temperature resistance. Table 3 summarizes its primary physical and mechanical properties at room temperature. Before additive manufacturing, the powder underwent vacuum drying at 120 °C for 2 h. The scanning strategy employed during deposition is illustrated by the blue lines in Figure 1.

### 2.2. Preparation of Test Samples

**Figure 2:**
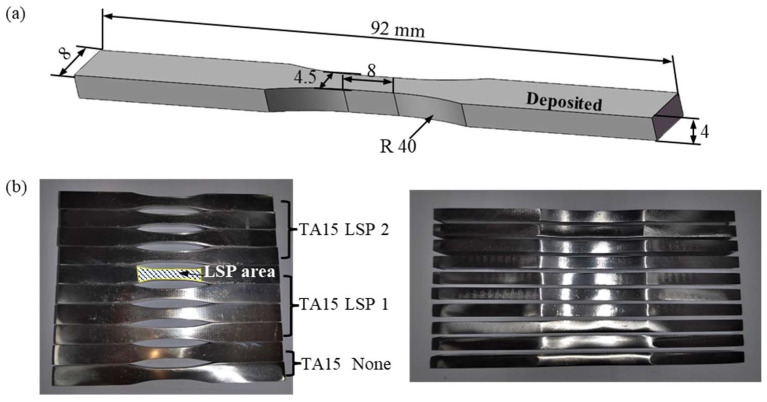
Fatigue Specimen Images: (**a**) Three-dimensional model and dimensions of the fatigue specimen, (**b**) Front and side views of the specimen after surface strengthening, along with the LSP region.

The manufacturing process for fatigue specimens of additive-processed TA15 titanium alloy primarily involves wire electrical discharge machining (WEDM). First, fatigue specimens were obtained from layered rectangular blocks using a wire cutting machine (AQ 900 L, Sodick, Yokohama, Japan). Figure 2 shows the geometry and dimensions of the fatigue specimen prior to LSP surface strengthening. Both front and back surfaces of the wire-cut specimen were ground and polished to minimize fatigue life errors caused by surface defects. The laser beam parameters employed during the LSP process were set as follows: wavelength 1064 nm, pulse duration 8 ns, single-pulse energy 1 J, spot size 2 mm, pulse repetition frequency 2 Hz, and 50% overlap between adjacent spots in the scanning path. Black tape was applied to the specimen surface as an absorption layer. LSP treatment was applied to the central region of each specimen, with both front and back sides processed. Surface strengthening of the titanium alloy was performed using 1 J of energy. Both single-strengthening and double-strengthening treatments were conducted. The design included two untreated samples, four samples subjected to one LSP impact, and four samples subjected to two LSP impacts.

### 2.3. Experimental Procedures

Rotary bending fatigue tests were conducted on TA15 titanium alloy specimens using a tension-compression fatigue testing machine (DURATT-D50, China) to evaluate their fatigue life. Test conditions were as follows: frequency 5000 r/min, stress ratio R = −1, maximum stress σmax = 300 MPa, ambient atmosphere. Specimens were secured at both ends of the testing machine using specialized fixtures to ensure consistent load application positions. Four test groups were established: untreated specimens (TA15 AR), specimens subjected to one laser shock peening (TA15 LSP-1), and specimens subjected to two LSP cycles (TA15 LSP-2).

Using wire-cut EDM technology, the fatigue fracture surface was completely excised (a 5 mm region below the fracture point). First, to investigate the influence on fatigue crack initiation behavior, the microstructure of the fatigue fracture surface was observed and analyzed using scanning electron microscopy (SEM, JSM-7800F, Akishima City, Japan). Subsequently, the surface of the cut fracture specimen—specifically the LSP-reinforced surface—was subjected to mild mechanical polishing. This was followed by etching with a titanium alloy-specific metallographic etchant (HF:CH_3_COOH:H_2_O = 1:1:4) for approximately 20 s to clearly reveal its surface microstructure.

**Figure 3:**
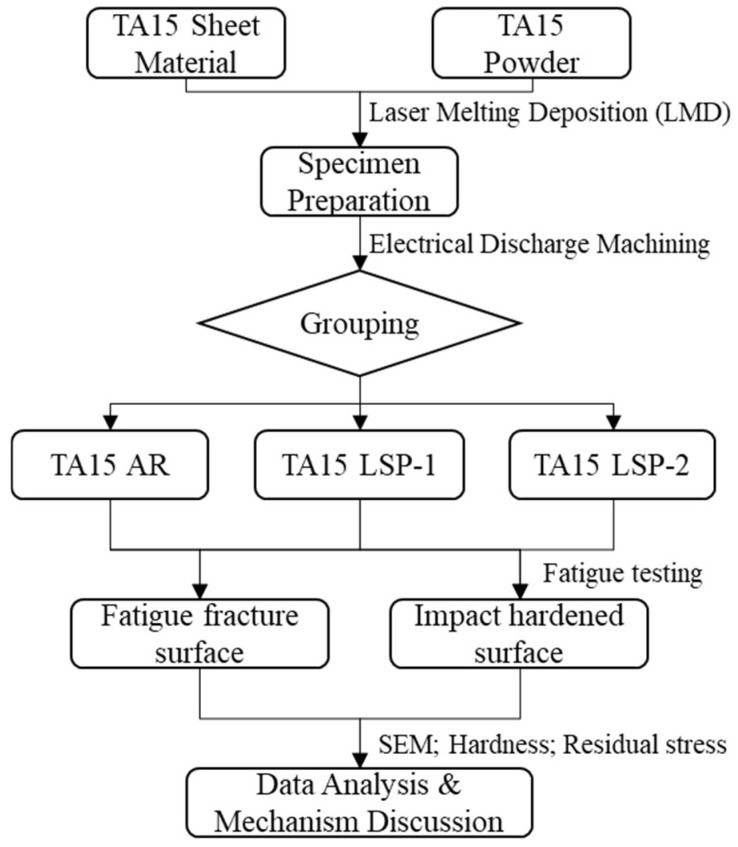
Sample Testing Flowchart.

Surface residual stresses in TA15 titanium alloy specimens were tested using a Bruker D8 Advance X-ray diffractometer (D8, Billerica, Germany). The microhardness of the LSP impact surface on all titanium specimens was measured using a THV-1 micro Vickers hardness tester (Changchun, China). The test load was 500 gf, and the specimen was removed after 15 s. Hardness points were tested within the LSP-strengthened zone at 5 mm intervals until no further strengthened area was present. During measurement, five readings were taken at each point, and the average hardness value at each point was recorded as the final hardness value. The flowchart from sample preparation to batch testing is shown in Figure 3.

## 3. Results and Discussion

### 3.1. Effect of LSP Count on Fatigue Life

**Figure 4:**
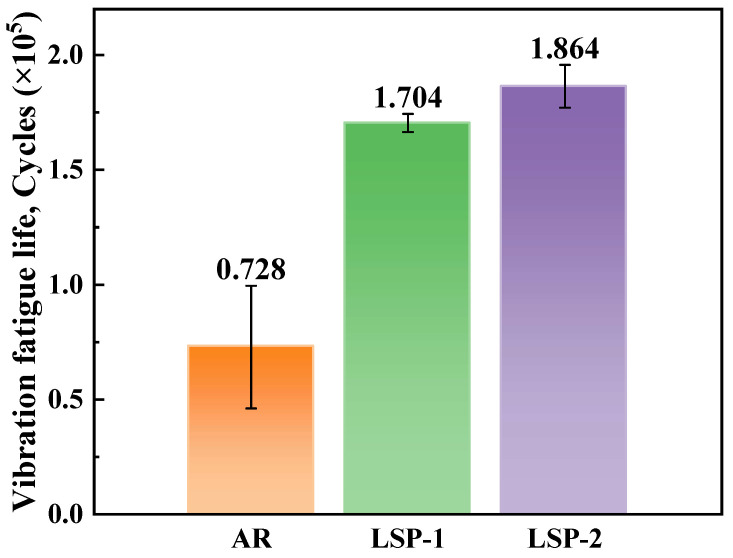
Life Diagram for TA15 AR, TA15 LSP-1, and TA15 LSP-2 Materials.

Figure 4 shows the 3D bar chart of fatigue life for all fatigue specimens. It is clearly visible that the fatigue life of the untreated TA15 material is very low, fracturing after only 72.82 thousand cycles. In contrast, the TA15 LSP-1 material withstands 170.40 thousand cycles under low-stress loading, achieving 2.34 times the fatigue life of the TA15 AR material. Furthermore, materials subjected to two LSP treatments exhibited further enhanced fatigue life. The fatigue cycle count of TA15 LSP-2 increased by 9.34% compared to TA15 LSP-1 and by 155.97% compared to TA15 AR. This indicates that TA15 LSP-2 withstands 2.56 times more tensile-compressive fatigue cycles than the standard TA15 AR material. It is clearly observed that LSP treatment of the material surface does enhance its fatigue life, and the effect of two impact treatments is superior to that of a single treatment.

### 3.2. Fatigue Fracture Toughness Analysis and Fracture Mechanism

**Figure 5:**
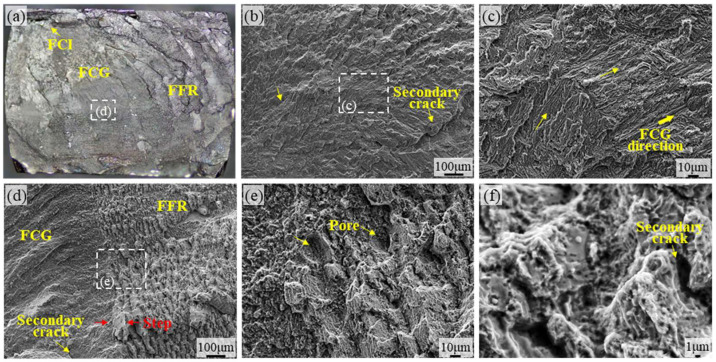
Fatigue fracture surface micrographs of TA15 AR prepared by LMD: (**a**) Overall morphology of the TA15 AR fracture surface, (**b**,**c**) SEM micrographs of the FCG region on the TA15 AR fatigue fracture surface, (**d**–**f**) SEM micrographs of the FFR region on the TA15 AR fatigue fracture surface.

Figure 5 shows the fatigue fracture surface of TA15 AR titanium alloy prepared by LMD. The fatigue crack initiation (FCI) region refers to the location where cracks first form, typically occurring at surface defects in the material. Figure 5a displays the overall fracture morphology of TA15 AR. The FCI region is observed at the material boundary, with the indented area indicating that FCI originated from the expansion of pre-existing pores within the material. Under cyclic tensile-compressive external loading, the region where the crack propagates outward from its initiation point is termed the fatigue crack growth (FCG) region. Figure 5b,c reveal numerous fine, smooth microfacets in the FCG zone, indicating that fatigue fracture involves intergranular fracture components. The fast fracture region (FFR) represents the area where material fractures instantaneously when unable to withstand sustained external forces. Figure 5a shows a large FFR area exhibiting a stepped morphology with ductile dimples and shear lips, characteristic of typical ductile fracture behavior. Figure 5e,f reveal potential defects such as porosity or incomplete fusion in the material during additive manufacturing, resulting in significant voids at the fatigue fracture surface. Secondary microcracks are observable across all fracture regions, a characteristic feature of tensile–compressive fatigue.

**Figure 6:**
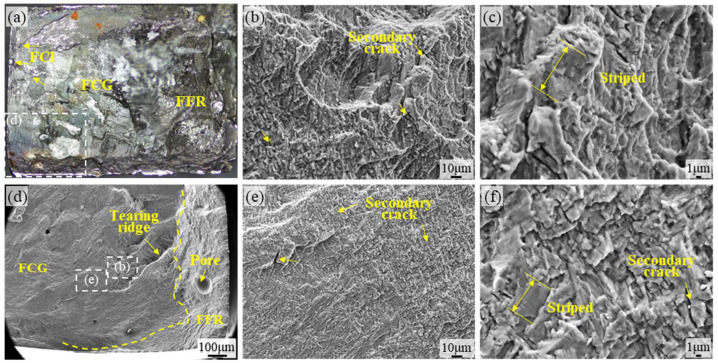
SEM micrographs of the TA15 LSP-1 fatigue fracture surface: (**a**) Overall morphology of the TA15 LSP-1 fracture surface, (**b**,**c**) SEM micrographs of the FFR region on the TA15 LSP-1 fatigue fracture surface, (**d**) SEM micrograph of the boundary region on the TA15 LSP-1 fracture surface, (**e**,**f**) SEM micrographs of the FCG region on the TA15 LSP-1 fatigue fracture surface.

Fatigue testing was conducted on the TA15 LSP-1 specimen. The overall fracture morphology is shown in Figure 6a. Although FCI appeared at surface defects, subsurface FCI also formed. Figure 6b shows the SEM morphology near the FFR region, revealing an extremely uneven fracture surface with secondary cracks visible. Figure 6d shows internal defects inherent to the material itself, which did not serve as crack initiation sites during fatigue testing. Concurrently, the fracture surface exhibits a morphology where ductile dimples coexist with tearing ridges, indicating that the material retained a degree of toughness at the point of ultimate fracture. Figure 6e shows the microstructure of the FCG zone at the fracture surface of TA15 LSP-1, exhibiting a flat and directional morphology. Figure 6c,f reveal a clear fatigue pattern with a relatively fine and uniform distribution, accompanied by a small number of secondary cracks. This indicates that cracks propagate along stress concentration zones under cyclic loading, and the residual compressive stress introduced by laser shock effectively delays the crack propagation rate. Compared to the un-hardened specimen, LSP-1 treatment shifted crack initiation from the surface to the subsurface layer [30] and significantly reduced the number of secondary cracks. This further demonstrates that laser shock peening effectively suppresses early crack initiation and propagation triggered by surface defects by refining grain size and introducing a compressive stress field, thereby enhancing the material’s fatigue performance.

**Figure 7:**
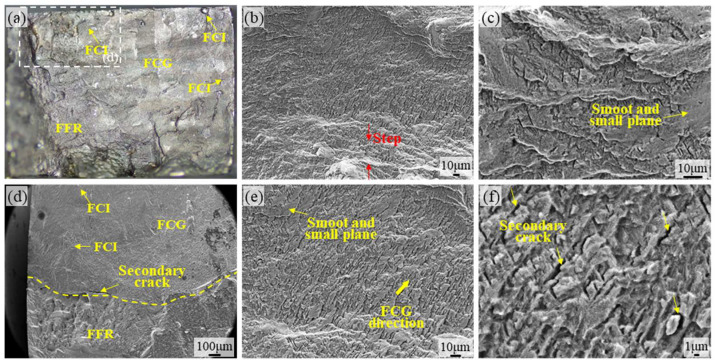
SEM micrographs of the TA15 LSP-2 fatigue fracture surface: (**a**) Overall morphology of the TA15 LSP-2 fracture surface, (**b**,**c**) SEM micrographs of the FFR region on the TA15 LSP-2 fatigue fracture surface, (**d**) SEM micrograph of the boundary region on the TA15 LSP-2 fracture surface, (**e**,**f**) SEM micrographs of the FCG region on the TA15 LSP-2 fatigue fracture surface.

Figure 7a shows the SEM micrograph of the fatigue fracture surface of the TA15 LSP-2 specimen. It can be observed that the FCI, similar to TA15 LSP-1, migrated from the surface to the subsurface layer. This indicates that LSP introduced compressive stress to the surface, reducing surface roughness and decreasing the number of initiation zones. In Figure 7b,c,e,f, the fatigue striations at the FCI region are fine and uniformly spaced, accompanied by distinct secondary cracks and numerous smooth microfacets. This indicates an increased proportion of intergranular fracture during tensile-compressive fatigue in the TA15 LSP-2 material. Figure 7d shows the coexistence state diagram of FCE and FFR, with the FFR region being relatively small overall. Ultimately, the FFR exhibits typical ductile dimple morphology, confirming the material retains good toughness. No pronounced ridge-like features were observed, and the fracture surface appears slightly smoother than that of TA15 LSP-1. Comprehensive analysis indicates that secondary strengthening treatment further optimizes the compressive stress distribution and microstructure at the surface layer, thereby more effectively enhancing the fatigue resistance of the TA15 titanium alloy.

### 3.3. Effect of LSP Frequency on the Surface Integrity of TA15

**Figure 8:**
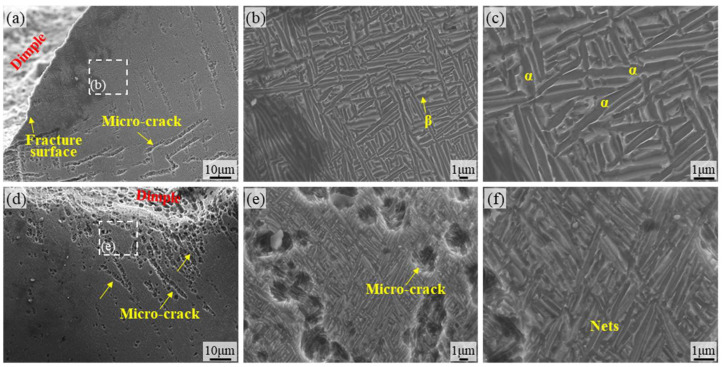
SEM micrographs of the TA15 AR specimen surface: (**a**,**d**) SEM micrographs of the TA15 AR surface region; (**b**,**c**) microstructural morphology within the region shown in (**a**); (**e**,**f**) microstructural morphology within the region shown in (**d**).

Figure 8 provides microstructural images of the surface of the TA15 AR specimen. Figure 8a,d reveal microcracks originating from the surface along the fracture edge under fatigue loading. These cracks primarily nucleate and propagate along grain boundaries of the primary α phase (equiaxed α) or α/β phase interfaces, demonstrating the vulnerability of grain boundaries under cyclic stress. The high-magnification images in Figure 8e,f reveal the meandering characteristics of crack paths, whose propagation direction is significantly influenced by microstructural anisotropy. Deflection occurs particularly at the boundaries of α-bundles or lamellar β-crystalline domains, directly reflecting the inhibitory effect of the basket-weave structure’s anisotropy on crack propagation. Additionally, localized plastic deformation traces are present in the crack tip region, while crack branching reflects the combined effects of multi-directional stress states and microstructural crack-stopping mechanisms. Figure 8b,c show the micrographs of the crack-free regions, where the titanium alloy exhibits a typical basket-weave structure. Based on the typical microstructure of additively manufactured TA15, the presence of molten pool boundaries or porosity-like defects near the surface further promotes microcrack nucleation.

**Figure 9:**
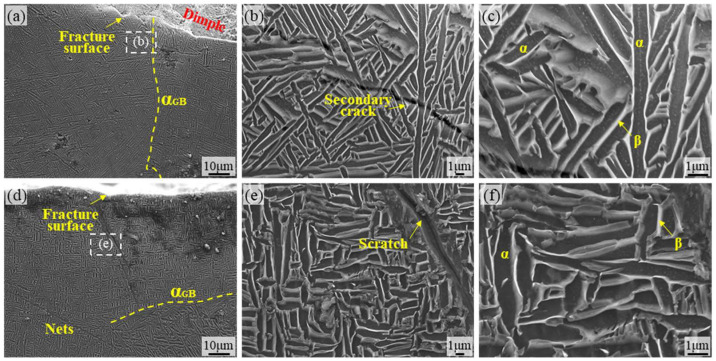
Microstructural micrographs of the fatigue fracture side surface of the TA15 LSP-1 specimen: (**a**,**d**) SEM micrographs of the TA15 LSP-1 surface region; (**b**,**c**) microstructural morphology within the region shown in (**a**); (**e**,**f**) microstructural morphology within the region shown in (**d**).

Figure 9 shows the surface microstructure of TA15 LSP-1. No obvious dense microcracks appeared on the material surface, likely due to the introduction of slight compressive stress by the LSP treatment, which improved surface quality. Distinct grain boundary structures are observable in Figure 9a,d. Meanwhile, Figure 9c,f reveal that α microstructure predominantly consists of elongated lamellar structures, with β microstructure growing around the α microstructure. Its microstructure resembles that of TA15 AR, consistent with the limited number of laser-assisted strengthening cycles, low energy input, and insufficient energy per LSP to induce significant microstructural remodeling. Large secondary cracks and defects are visible in the regions shown in Figure 9b,e. This is because LSP treatment improves surface quality, reducing the impact of fatigue cycles across the entire material area and significantly decreasing the number of secondary cracks generated. Furthermore, the growth direction of secondary cracks is predominantly intergranular fracture, with a small portion exhibiting transgranular fracture. This aligns with the observation of a small number of smooth microfacets noted in fracture analysis.

**Figure 10:**
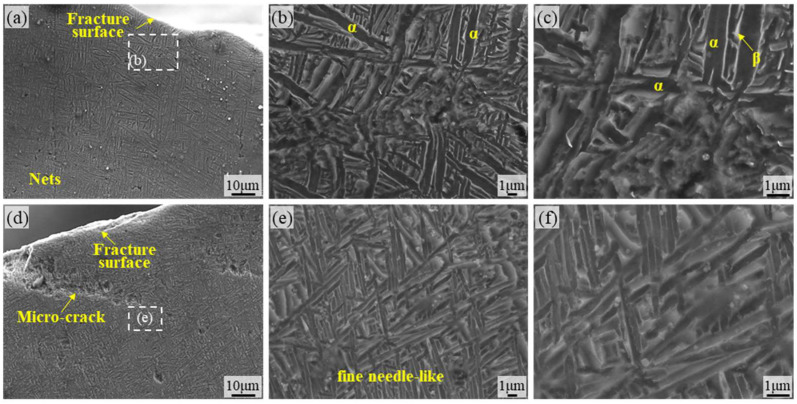
Microstructure of the fatigue fracture surface of the TA15 LSP-2 specimen: (**a**,**d**) SEM micrographs of the surface region of the TA15 LSP-2 specimen; (**b**,**c**) microstructural morphology within the region shown in (**a**); (**e**,**f**) microstructural morphology within the region shown in (**d**).

Figure 10 shows the surface SEM micrograph of TA15 LSP-2. It can be clearly observed that the microstructure is significantly refined compared to that of TA15 AR and TA15 LSP-1 materials. The lamellar structure has been refined into acicular martensite, resulting in a mixed microstructure where lamellar α-phase coexists with acicular martensite. This indicates that secondary LSP strengthening causes significant changes to the surface microstructure of the material and exhibits a pronounced grain refinement effect [33]. Grain refinement directly leads to a significant increase in the number of grain boundaries. This not only enhances material strength (Hall-Petch effect) but also provides more obstacles for crack propagation, making the crack propagation path more tortuous and consuming more energy. Furthermore, Figure 10a,d reveal no obvious secondary cracks in the fracture zone of the TA15 LSP-2 specimen, with smooth fracture edges and a clearly defined fracture location. This directly demonstrates that a refined and uniform microstructure can effectively suppress the initiation of secondary cracks, thereby enhancing fatigue life. Figure 10b–f reveals the coexistence of lamellar α structure and needle-like martensite in the microstructure of TA15 LSP-2 material. It is believed that increasing the number of LSP strengthening cycles can fully transform the microstructure into fine needle-like martensite [29], at which point the material exhibits its highest resistance to fatigue cycle fracture.

### 3.4. Hardness and Residual Stress Test Results

**Figure 11:**
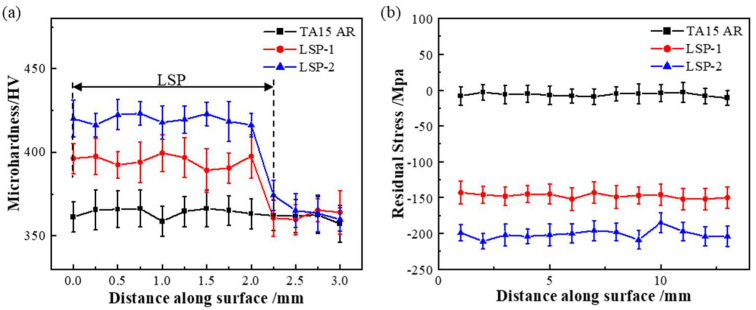
Fatigue specimen test results: (**a**) Surface hardness data diagram, (**b**) Surface residual stress data diagram.

Most cracks in engineering materials originate from the surface layer. Increasing microhardness can suppress crack initiation and enhance the fatigue performance of treated materials [34]. Figure 11a shows the microhardness distribution on the surfaces of specimens subjected to different numbers of laser impact treatments. It can be observed that the untreated TA15 specimen exhibits an average surface microhardness of 360 HV. Specimens subjected to one and two laser shock treatments demonstrate surface microhardness values of 390.6 HV and 412.3 HV, respectively, representing increases of 8.51% and 14.53% compared to the untreated specimen. The increase in surface hardness induced by LSP is primarily attributed to shock-induced plastic deformation and grain refinement effects. As the number of impacts increases, cumulative deformation leads to elevated dislocation density, causing the α-phase lamellar structure to gradually transform into finer acicular martensite, thereby further enhancing hardness.

Introducing high compressive residual stresses (CRS) on material surfaces can effectively reduce the effective stress intensity factor range at crack tips under actual cyclic loading [35]. This suppresses microcrack propagation during the early fatigue stage and improves surface microdefects [36], preventing their development into fatigue crack initiation sites. Figure 11b shows the CRS distribution on the surfaces and along the depth direction of LP-treated specimens. At the same depth, the CRS value continuously increases with the number of laser impacts [37]. The results indicate that the CRS values for TA15 AR, TA15 LSP-1, and TA15 LSP-2 are −5 MPa, −145 MPa, and 183 MPa, respectively. The formation of residual compressive stress results from compressive plastic strain in the surface layer material under shock wave action, with multiple impacts enhancing both the depth and magnitude of the compressive stress layer. These combined improvements in surface layer properties influence fatigue behavior, delaying crack initiation and inhibiting its propagation. The compressive stress present on the original specimen surface may be attributed to the slight pressure applied during surface polishing. In contrast, the compressive stress in laser shock-hardened specimens results from the significant impact force generated when the laser strikes the specimen surface. A higher number of laser impacts leads to greater compressive stress and a deeper influence layer.

## 4. Conclusions

This study systematically investigated the influence of LSP cycles on the performance and fracture mechanism evolution of LMD-processed TA15 titanium alloy under low-stress fatigue loading. The main conclusions are as follows:

(1) LSP treatment significantly enhances the fatigue life of the material. The fatigue life of the single-stage strengthened (TA15 LSP-1) specimen reached 170,400 cycles, which is 2.34 times that of the TA15 AR specimen. The fatigue life of the double-stage strengthened (TA15 LSP-2) specimen further increased to 2.56 times that of the AR specimen, indicating that the strengthening effect is cumulative.
(2) LSP promotes the migration of fatigue crack initiation from the surface to the subsurface, improves crack propagation behavior, results in finer and more uniform fatigue striations, reduces secondary cracking, and effectively delays the crack growth rate.
(3) LSP optimized the fracture mechanism by preserving the dimple characteristics in the final fracture zone while reducing the proportion of intergranular fracture. The fracture surface morphology of TA15 LSP-2 specimens exhibited a smoother profile, further enhancing fracture resistance.
(4) With increasing LSP cycles, the surface microstructure becomes finer. Specimens treated with TA15 LSP-2 exhibit a coexisting microstructure of lamellar α and acicular martensite, demonstrating significant grain refinement that forms the microscopic basis for improved fatigue performance.
(5) Mechanical property testing indicates that LSP treatment significantly enhances surface material properties by inducing plastic deformation in the surface layer. With increasing LSP cycles, surface microhardness rose from 360 HV in the untreated state to 390.6 HV (TA15 LSP-1, an 8.51% increase) and 412.3 HV (TA15 LSP-2, a 14.53% increase), while surface residual compressive stresses reached -145 MPa and -183 MPa, respectively. This synergistic effect of surface hardening and compressive stress effectively suppresses fatigue crack initiation and propagation, constituting a key mechanism by which LSP enhances the fatigue performance of TA15 titanium alloy.

In summary, laser shock peening effectively improves the fatigue performance of additively manufactured TA15 titanium alloy through the synergistic effects of introducing residual compressive stress and refining the surface microstructure. The results indicate that secondary strengthening demonstrates more pronounced effects in enhancing material surface integrity, optimizing crack behavior, and improving fracture mechanisms, providing important theoretical and experimental basis for enhancing the fatigue performance of additively manufactured titanium alloy components.

## Figures and Tables

**Table 1:** Fixed Manufacturing Parameters for the LMD Process.

Process Parameters	Parameter Value
Scanning speed (mm/s)	10
Laser power (W)	2400
Laser spot diameter (mm)	4
Overlap ratio (%)	50
Interlayer height (mm)	0.6
Protective gas (L/min)	15
Types of shielding gases	Ar

**Table 2:** Chemical Composition of TA15 Titanium Alloy.

Component (wt.%)	Fe	C	Al	V	Zr	Mo	Ti
TA15	≤0.25	≤0.10	5.5–7.1	1.8–3.7	1.5–2.5	0.5–2.0	Bal.

**Table 3:** Physical and Mechanical Properties of TA15 Alloy at Room Temperature.

Material	Tensile Strength $\sigma_{b}$/MPa	Yield Strength $\sigma_{0.2}$/MPa	Elongation δ/%	Elasticity Modulus *E*/GPa	Density ρ/(kg·m^−3^)	Poisson’s Ratio *υ*
TA15	902.04	434.52	8.50	115.79	4.45	0.38

## Data Availability

The original contributions presented in this study are included in the article. Further inquiries can be directed to the corresponding authors.

## References

[1] Liu Z., He B., Lyu T., Zou Y. (2021). A Review on Additive Manufacturing of Titanium Alloys for Aerospace Applications: Directed Energy Deposition and Beyond Ti-6Al-4V. J. Miner. Met. Mater. Soc..

[2] Wang D., Geng X., Liu H., Guo D., Lu X., Zheng D., Xu Y., Guan M., Zhang Y., Du B. (2026). Effect of heat treatment on microstructure and mechanical properties of electron-beam powder bed fused TiB w/TA15 composite. Mater. Sci. Eng. A.

[3] Ning J., Zhao J., Zhang L. (2025). Effect of laser offset on microstructure and mechanical properties of Nb521/TA15 dissimilar joints. J. Mater. Res. Technol..

[4] Yang Y., Yan S., Qu S., Yi M., Chen Z., Zhou T., Xu C. (2025). Microstructure of titanium alloy in additive/subtractive hybrid manufacturing: A review. J. Alloys Compd..

[5] Tian Q.M., Yang Y., Tan Y.B., Xiang S., Zhao F., Ji X.M., Huang G. (2025). Synergistically enhancing the strength and ductility of TA15 titanium alloy through hot rolling and short-time annealing. J. Alloys Compd..

[6] Yang X., Shen D., Wang Q., Su Y., Liu Y., Guo Z., Xiong R., Meng Y., Feng Z., Hao S. (2025). Microstructure control and high-temperature mechanical response of TA15 titanium alloy fabricated by selective laser melting. Mater. Today Commun..

[7] Tanwar R.S., Jhavar S. (2024). Ti based alloys for aerospace and biomedical applications fabricated through wire + arc additive manufacturing (WAAM). Mater. Today Proc..

[8] Liu S., Shin Y.C. (2019). Additive manufacturing of Ti6Al4V alloy: A review. Mater. Des..

[9] Ratnala D.C., Hanning F., Joshi S., Andersson J. (2025). The parametric investigation and microstructural characterization of laser directed energy deposited NiCrAlY powder. J. Mater. Res. Technol..

[10] Hua B., Yin J., Wang B., Lu Y., Zhan W., Huang K., Han B., He B., Zhang Q. (2025). Microstructure evolution and performance improvement of 42CrMo steel repaired by an ultrasonic rolling assisted laser directed energy deposition IN718 superalloy. J. Alloys Compd..

[11] Deng H., Chen Y., Jia Y., Pang Y., Zhang T., Wang S., Yin L. (2021). Microstructure and mechanical properties of dissimilar NiTi/Ti6Al4V joints via back-heating assisted friction stir welding. J. Manuf. Process..

[12] Shen Y., Wang L., Li L., Kong D., Ma R., Liu L., Li P. (2025). Microstructure evolution of 2205 duplex stainless steel (DSS) and inconel 718 dissimilar welded joints and impact on corrosion and mechanical behavior. Mater. Sci. Eng. A.

[13] Zhou S., Qian Z., Zhang Z., Bai X. (2025). A novel fatigue life prediction method for the laser deposition repaired TA15 component with annealing heat treatment. Eng. Fail. Anal..

[14] Sun P.F., Qu C.X., Duan C.F., Li F.L., Li X.Q., Qu S.G. (2025). Explore the nature of gradient deformation layer constructed by high-density electric pulse assisted ultrasonic nanocrystalline surface modification for Ti6Al4V alloy. J. Alloys Compd..

[15] Zhao T.Y., Wang Z.Y., Wu H., Wu C.L., Zhang C.H., Zhang S., Chen H., Zhang D. (2025). A hybrid laser surface modification technique: Microstructural and property regulation mechanisms of titanium alloy via laser shock forging. Mater. Charact..

[16] Edwards P., Ramulu M. (2014). Fatigue performance evaluation of selective laser melted Ti-6Al-4V. Mater. Sci. Eng. A.

[17] Tang D., He X., Wu B., Dang L., Xin H., Li Y. (2024). A fatigue life prediction approach for porosity defect-induced failures in directed energy deposited Ti-6Al-4V considering crack growth environment. Int. J. Fatigue.

[18] Chen Z.B., Cui X.L., Yu M.R., Jiang G.R., Liu F.Q., Yang X.Y., Chen J. (2025). Microstructure evolution and strengthening mechanisms of laser directed energy deposited TA15 titanium alloy with synchronous ultrasonic impact. J. Alloys Compd..

[19] Tan L., Tang W., Wang M., Zhang Y., Yao C. (2025). Studies on surface integrity and fatigue performance of Ti-17 alloy induced by ultrasonic surface rolling process. Surf. Coat. Technol..

[20] Warzanskyj W., Angulo I., Cordovilla F., Díaz M., Porro J.A., García-Beltrán A., Cabeza S., Ocaña J. (2023). Analysis of the thermal stability of residual stresses induced in Ti-6Al-4 V by high density LSP treatments. J. Alloys Compd..

[21] Guadalupe M., Bravo A., Gomez-rosas G., Morales M., Munoz-martin D., Moreno-labella J.J., Lopez J.M.L., Galvan J.G.Q., Rubio-Gonzalez C., Rodriguez F.J.C. (2025). Effects of Laser Shock Processing on the Mechanical Properties of 6061-T6 Aluminium Alloy Using Nanosecond and Picosecond Laser Pulses. Materials.

[22] Li P., Wang S., Wu J., Shen Y., Dong Z., Xu H. (2026). Ultrasonic surface rolling process for improving fatigue crack propagation resistance of laser-clad TC4-TA15 titanium alloy. Surf. Coat. Technol..

[23] Wu B., Huang J., Yang G., Ren Y., Zhou S., An D. (2022). Effects of ultrasonic shot peening on fatigue behavior of TA15 titanium alloy fabricated by laser melting deposition. Surf. Coat. Technol..

[24] Kumar S., Chattopadhyay K., Singh V. (2020). Optimization of the Duration of Ultrasonic Shot Peening for Enhancement of Fatigue Life of the Alloy Ti-6Al-4V. J. Mater. Eng. Perform..

[25] Tang K., Zhu J., Li Z., Chen S., Zhang Y., Yan Y., Hao Q., Guo B., Zhong F., Chen W. (2025). Study on the Effect of Laser Shock Angle on Surface Integrity and Wear Performance of H13 Steel. Lubricants.

[26] Li P., Shen Y., Hua Z., Wu J., Wang S., Zhang J. (2025). Improving the corrosion resistance and wear resistance of Carbon Steel by laser shock peening and shot peening. Tribol. Int..

[27] Zheng K., Zheng F., Lin J., Ren Z., Lin Y. (2025). Utilization of red mud as copper substitute in eco-friendly resin-based brake composites: Performance evaluation and machine learning prediction. Tribol. Int..

[28] Wang C., Zhang Q., Xu L., Li L., Deng W., Zhang T., Liu Y., Xu G., Liu L., Li P. (2025). Obvious corrosion resistance of 2Cr13 martensitic stainless steel subjected to thermo-mechanical laser shock peening and subsequent slight polishing. Chin. J. Aeronaut..

[29] Fang X., Liu C., Li K., Feng J., Qiao R., Zhou J., Yang J., Chang T., Huang K., Lu B. (2025). Improved properties of wire-arc directed energy deposited Al—Mg alloy through laser shock peening. J. Mater. Res. Technol..

[30] Kljestan N., Balos S., Pecanac M., McWilliams B.A., Knezevic M. (2025). Effects of laser shock peening on fatigue performance of an ultrahigh-strength low-alloy steel fabricated via laser powder bed fusion. J. Mater. Res. Technol..

[31] Pradeep Kumar S., Radhika N., Dinesh Babu P., Ram Prabhu T., Gautam J., Chakkravarthy V. (2025). Evaluation of high-temperature wear behaviour of selective laser melted 17-4 PH stainless steel through laser shock peening. J. Mater. Res. Technol..

[32] Qu S., Sha Y., Hou Y., Wang J., Li F., Li X. (2025). Effect of Laser Shock Peening on High-Cycle Fatigue Performance and Residual Stress in DH36 Welded Joints. Materials.

[33] Bricín D., Jansa Z., Kaufman J., Špirit Z., Fulín Z., Strejcius J. (2025). Effect of laser shock peening on the microstructure of GX4CrNi13–4 martensitic stainless steel. J. Manuf. Process..

[34] Ao N., Liu D., Zhang X., Liu C., Yang J., Liu D. (2019). Surface nanocrystallization of body-centered cubic beta phase in Ti–6Al–4V alloy subjected to ultrasonic surface rolling process. Surf. Coat. Technol..

[35] Aoki T., Hayama M., Kikuchi S., Komotori J. (2025). Peening methods for AISI 4140 steel to induce stable compressive residual stress against cyclic axial loading. Int. J. Fatigue.

[36] Zhang H., Li X., Li C., Cao S., Liu C. (2025). Hybrid treatment of shot peening and vibratory finishing for fatigue enhancement in laser powder bed fused 304L steel. J. Mater. Process. Tech..

[37] Xu P., Chen C., Sun H., Zhang Y. (2025). Effect of laser shock peening on residual stress of 316L stainless steel laser metal deposition part. J. Mater. Res. Technol..

